# Clinical and epidemiological characteristics and individual experiences of illness in men with COVID-19: mixed method study

**DOI:** 10.1590/1516-3180.2021.0747.R1.22022022

**Published:** 2022-08-29

**Authors:** Andressa Reis de Sousa Vilas Boas, Daniel Gomes Santos, Jules Ramon Brito Teixeira, Luciano Garcia Lourenção, José Luís Guedes dos Santos, Richardson Augusto Rosendo da Silva, Ana Raquel Batista de Carvalho, Álvaro Francisco Lopes de Sousa, Anderson Reis de Sousa

**Affiliations:** I Nurse, Municipal Health Department of Quixabeira, Bahia, Brazil.; II Nurse, Municipal Health Department of Quixabeira, Bahia, Brazil.; III PhD. Professor, Postgraduate Program in Collective Health, Universidade Estadual de Feira de Santana (UEFS), Feira de Santana (BA), Brazil.; IV PhD. Nurse and Full Professor, Postgraduate Nursing Program, Universidade Federal do Rio Grande (FURG), Rio Grande (RS), Brazil.; V PhD. Assistant Professor, Postgraduate Nursing Program, Universidade Federal de Santa Catarina (UFSC), Florianópolis (SC), Brazil.; VI PhD. Professor, Postgraduate Nursing Program, Universidade Federal do Rio Grande do Norte (UFRN), Natal (RN), Brazil.; VII MSc. Doctoral Student, Postgraduate Nursing Program, Universidade Federal do Piauí (UFPI), Teresina (PI), Brazil.; VIII RN, PhD. Assistant Professor, Nursing Department, Centro Universitário UNINOVAFAPI, Teresina (PI), Brazil, and Reseacher, Global Health and Tropical Medicine (GHTM), Instituto de Higiene e Medicina Tropical (IHMT), Universidade Nova de Lisboa, Lisboa, Portugal.; Global Health and Tropical Medicine, Instituto de Higiene e Medicina Tropical, Universidade Nova de Lisboa, Lisboa, Portugal; IX PhD. Professor, Postgraduate Nursing Program, Universidade Federal da Bahia (UFBA), Salvador (BA), Brazil.

**Keywords:** Coronavirus infections, Pandemics, Men’s health, Epidemiology, SARS-CoV-2, Coronavirus, Coronavirus disease 2019 virus, Epidemics of infectious disease, Health profile, Social epidemiology

## Abstract

**BACKGROUND:**

Since the beginning of the coronavirus disease 2019 (COVID-19) pandemic, studies have shown that this disease has affected the male population on a significant scale in various parts of the world, making men one of the main risk groups.

**OBJECTIVE:**

To analyze the clinical and epidemiological characteristics and experiences of illness in men with COVID-19.

**DESIGN AND SETTING:**

A mixed sequential-explanatory study with cross-sectional and exploratory-descriptive approaches.

**METHOD:**

Data was collected from a small municipality located in the central-north region of the state of Bahia, Brazil. Primary quantitative data was extracted from compulsory notification forms from 598 men. Qualitative data from individual interviews of 30 men was analyzed by the Discourse of the Collective Subject method.

**RESULTS:**

The findings identified the characterization of reports of suspected and confirmed cases of COVID-19 in men, the organization of the healthcare system, and strategies for the control and combat of COVID-19 directed towards the men of the investigated municipality. They revealed the clinical characteristics based on the collective discourse of men with COVID-19.

**CONCLUSION:**

In men, the individual experience of disease explicitly explains the clinical markers of COVID-19 expressed by the self-reported syndromic approach. Additionally, this understanding also explains the behaviors observed in their search for health care, as well as the adoption of prevention and control measures and therapies recommended by health professionals.

## INTRODUCTION

Coronavirus disease 2019 (COVID-19) has significantly affected the male population in various parts of the world.^1^ ^,^ ^2^ ^,^ ^3^ ^,^ ^4^ In countries such as Brazil, the number of cases of the disease in men varies according to municipality.^5^ ^-^ ^6^

In addition to being the most affected gender, men have the worst clinical outcomes. Among the population that progressed to a picture of severe acute respiratory syndrome (SARS) caused by COVID-19, until the epidemiological period from February 21st to 27th, 2021, 62,613 cases were registered, with 54.5% of these cases comprising men. The most affected age group from this population was between 60 and 69 years, with a total of 24,775 cases (21.6%). Regarding deaths caused by SARS due to COVID-19 in Brazil, during this period, 16,444 (54.3%) were men, with the most affected age group between 70 and 79 years old, with a total of 7,717 (25.5%).^7^

Several reasons have been suggested in the literature for the difference between sexes in the presentation of COVID-19, which indicate that being male is a risk factor for the new disease. Among these reasons, the influence of hegemonic patterns of masculinities was highlighted. This legitimizes the dominant position of men in society, which can contribute to the neglect of health care, and the disregard and disrespect for measures to prevent and control the transmission of SARS-CoV-2.^8^ ^,^ ^9^

There are also factors related to habits such as smoking, abusive consumption of alcohol and other drugs, sedentary lifestyle, and clinical conditions, such as the presence of chronic diseases which occur more often in men. Moreover, new causes have been recognized with the arrival of the pandemic, namely hormonal factors related to testosterone^10^ and generators of repercussions on sexual and reproductive health,^11^ genetic factors of chromosomal origin,^12^ and immunological factors^1^ ^,^ ^3^.

Another relevant aspect to be considered that has also been occurring in the context of the pandemic, is that a large proportion of the male population delays searching for healthcare services.^7^ ^,^ ^9^ This resistance to seeking healthcare is necessary for the early detection of COVID-19 infections and leads to increased underreporting and/or late compulsory notification. It also leads to the progression of infections into more severe and complex clinical situations. Moreover, one cannot lose sight of the aspects related to weaknesses in healthcare services and networks, limited healthcare human resources^13^ and the presence of barriers to access and sensitize the male population for health promotion and prevention of diseases and avoidance of injuries.^14^ ^,^ ^15^

When considering the presence of a gap in scientific knowledge in the difference in disease presentation based on sex and gender, and given the clinical and epidemiological context of men with COVID-19, the combination of quantitative and qualitative approaches used in this study is justified. A joint study may contribute to a broader interpretation of the investigated problem, providing better evidence for best practice care for men with COVID-19.

## OBJECTIVE

To analyze the clinical and epidemiological characteristics and experiences of illness in men with COVID-19.

## METHODS

### Research design

A mixed methods study, with sequential-explanatory type from a transverse and analytical study, and an exploratory-descriptive research with a qualitative approach. In this study design, the quantitative approach was developed first and had the greatest weight; that is, it is the priority stage of the research. Next, a qualitative approach of secondary weight and complementary character was developed. Quantitative data were extracted from COVID-19 compulsory notification forms. Individual interviews were conducted at the qualitative stage. Thus, the data collected in the quantitative stage led and directed the data collection of the qualitative stage,^15^ especially regarding the details of clinical characteristics and experiences of patients such as the recognition of signs and symptoms, perception of the disease, adherence to therapies and treatment, and apprehension of senses and meanings.

### Data collection period

The study population consisted of suspected cases of infection with the COVID-19 in men, notified to the municipal epidemiological surveillance, from February to December 2020.

### Selection criteria

Suspected cases of COVID-19 infection were individuals with an acute respiratory condition, characterized by at least two of the following signs and symptoms: fever (even if referred), chills, sore throat, headache, cough, runny nose, olfactory disorders, or taste disturbances.

The study excluded males who passed through the city, were suspected, or were assisted/notified during the data collection period.

### Sample

The quantitative sample consisted of 598 men, notified as suspected cases of SARS-CoV-2 infection from February to December 2020. The qualitative sample consisted of 30 men with a confirmed diagnosis of COVID-19 presenting with symptoms. The men included exclusively accessed the Municipal Center for Coping with COVID-19 and sought care. In addition, we sought participants who only accessed the service to ensure greater sample specificity, considering that it is a rural municipality, with habits, customs, and health behaviors that may have influenced the way they experienced the disease. The municipal health department, epidemiological surveillance sector, and participants agreed to access the data contained in the medical records.

### Data collection

Data were collected in the municipality of Quixabeira, Bahia, Brazil, from October to December 2020. During this period, the municipality had 598 notified cases, diagnosed using reverse transcription polymerase chain reaction (RT-PCR) (311 cases) or rapid tests (87 cases). At the end of that period, seven cases were still under monitoring, awaiting test results. Of these, 121 cases were positive: 77 (63.6%) were women and 44 (36.4%) were men. The municipality registered three cases of clinical admissions in a reference emergency care unit, one case of hospitalization requiring assistance in the intensive care unit, and no confirmed cases of death during the investigated period.

Data collection was conducted in two stages. The first - the quantitative stage - was based on data from the Municipal Epidemiological Surveillance, obtained via access to primary data from the Ministry of Health records of “compulsory notification forms of suspicious of coronavirus disease 2019 - COVID-19 Flu-like Syndromes.” The data were exported from the Google Forms application to an *Excel* spreadsheet, and manipulated for structuring the bank, checking for incompleteness and duplication. In the second stage - the qualitative stage - individual in-depth interviews were conducted with the participants.

The interviews were conducted by a single interviewer researcher and were audio-recorded in a single meeting, with an average duration of 30 minutes. They were previously scheduled under the support of the Epidemiological Surveillance service and the team of the COVID-19 Combat Center of the investigated municipality. Interviews adhered to health protocols and ensured the safety of the participants and the research team. Additionally, patients were later re-evaluated by a medical professional and given a diagnosis and cure for the disease. Participants were intentionally selected based on the indication of professionals from the Epidemiological Surveillance Service and the municipal COVID-19 Combat Center. An unstructured script was used for the following open questions: Tell us how you experienced the impairment caused by COVID-19? How does COVID-19 manifest clinically? This management was conducted using data surveys in the quantitative stage.

### Data processing and analysis

For data collection, notification forms were accessed from the health surveillance department after prior authorization. Then, data from the forms were read, organized, and encoded in a spreadsheet in Microsoft Excel application, version 16.0, developed by Microsoft (Redmond, Washington, United States), and submitted to processing in the Statistical Package for Social Sciences (SPSS) software, version 23.0, developed by the International Business Machines Corporation (IBM) (Nova York, United States).

After collecting the qualitative data, the narratives were read line by line, and the data were systematized and coded with the support of NVIVO12 software developed by QSR International (Melbourne, Australia). COREQ guidelines were followed to ensure the quality of the qualitative data. The analysis of narrative data was performed using the Discourse of the Collective Subject (DCS) technique,^16^ which facilitated raising the Key Expressions (KE) and the Central Ideas (CI) of collective representation of the research group on the phenomenon of patient experience with COVID-19.

DCS is a category of exposure to the results of qualitative research, with testimonies as raw material, in the form of one or more synthesis speeches written in the first person of the singular. This method consists of pointing out, from each answer, the KEs, which are the CIs of the discursive content expressed by the interviewees.^16^ The data described on the form of a Discourse-Synthesis, first person singular, represent the DCS of the men.

After the analysis of the quantitative and qualitative data, a combination of the data was conducted through the connection and integration of the results. Thus, additional information on the study objectives was identified.

Since this research was conducted in the context of the still-in-progress COVID-19 pandemic, ethical requirements in research were fulfilled, which involved biosafety to preserve the participants and researchers. The interviews were carried out with the researchers duly dressed in compliance with social distancing. The application of the Free and Informed Consent Term was associated with the dispensing of alcohol gel and disposable tissues to access the pens made available by the researchers.

The study was approved by the Research Ethics Committee (CEP) of the Universidade Federal da Bahia (UFBA) under Opinion No. 4,087,611 on June 15, 2020. Furthermore, we obtained authorization from the Municipal Health Department and Municipal Health Surveillance Department of Quixabeira to access notification forms and patient records.

## RESULTS

The first part of the results, derived from notifications, describes the clinical and epidemiological characteristics of men with COVID-19. The second part presents data from patient records, relating to clinical and epidemiological features and the control and combat strategies for COVID-19. Finally, the third source of data presents the clinical characteristics from the collective discourse of participants, retrieved from interview audio.

### Characterization of reports of suspected COVID-19 cases in men

This study included 515 reports of suspected COVID-19 infections in men living in the municipality. As shown in Table 1, most notifications were made by health services in the state itself (98.6%), predominantly men aged 25-59 years (61.9%), mixed race (57.5%), and those who did not work in healthcare (98.6%) **(** Table 1 **).**

**Table 1.** Sociodemographic characteristics of participants suspected of having coronavirus disease 2019 (COVID-19). Brazil, 2020-2021

Variables	n	%
Notifying state		
Bahia	508	98.6
Other states	7	1.4
Age group		
< 12 years	47	9.1
12 to 18 years	25	4.9
19 to 14 years	52	10.1
25 to 59 years	319	61.9
60 years or more	72	14.0
Race/color		
Mixed	296	57.5
White	85	16.5
Black	75	14.6
Yellow	49	9.5
Ignored	10	1.9
Health professional		
Yes	7	1.4
No	508	98.6

Source: Prepared by the authors. Research data.

**Figure 1.**
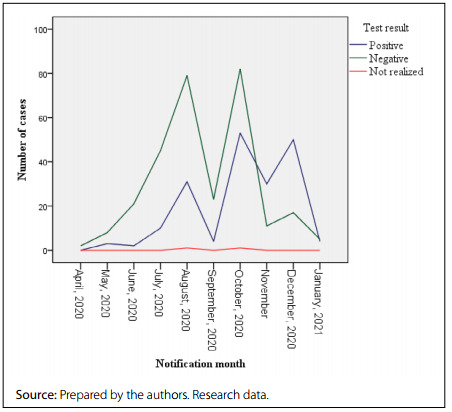
Distribution of suspected cases of coronavirus disease 2019 (COVID-19) in men, according to the month of notification and the result of the diagnostic test. Brazil, 2020-2021.

As shown in Figure 1, there was an increase in the number of notifications from April to August 2020, when the peak in the number of notified cases occurred. From then on, there was an oscillation in the number of suspected cases registered, with an increase during April to August 2020. The increase in the number of confirmed cases followed what happened with the notifications **(** Figure 1 **)**.

**Figure 2.**
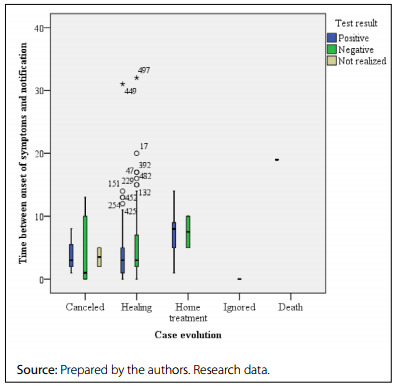
Distribution of suspected cases of coronavirus disease 2019 (COVID-19) in men, according to the days between symptom onset and notification, diagnostic test results and case evolution. Brazil, 2020-2021.

Data analysis showed that 20.6% of cases were notified eight days or more after the onset of symptoms, causing delays in the surveillance process and possibly in the medical care of these patients. As shown in Figure 2, there was a delay in notification among patients kept in home care or who died **(** Figure 2 **).**

**Figure 3.**
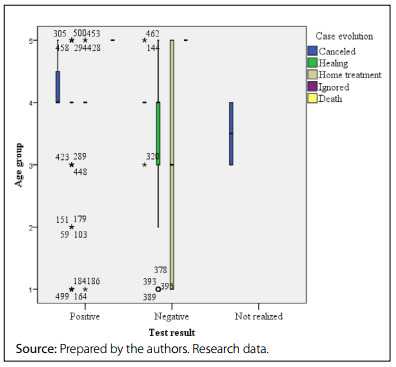
Distribution of suspected cases of coronavirus disease 2019 (COVID-19) in men, according to age group, diagnostic test result and case evolution. Brazil, 2020-2021.

The results also showed that death occurred in older men (≥ 60 years). The men kept in home care, who were negative for COVID-19, had an average age of 19-24 years. Reports of young teens and adult men who had confirmed COVID-19 infections predominantly progressed to cure **(** Figure 3 **)**.

The secondary Qual stage of the study involved data from 30 interviews with participants in the first quantitative stage, which was explained in the form of discourse synthesis. Its respective CIs were framed, theoretically, from the fields/variables that make up the “compulsory notification forms of suspicious of coronavirus disease 2019 - COVID-19 Flu-like Syndromes.”

Narrative qualitative data presented in the form of DCS of participants were retrieved to further investigate the clinical and epidemiological male health from the perception of users. The following illustrates a Discourse-Synthesis composed of a CI of representation of the collective as a whole regarding the clinical characteristics and experience of COVID-19.

### Discourse-synthesis: Clinical characteristics, from the collective discourse of men with COVID-19

The male collective discourse revealed that the clinical characteristics of COVID-19 are demarcated by the presentation of nonspecific symptoms. However, the disease presented its course with clinical characteristics of classic symptomatologic evolution, progressiveness, and worsening of the health status and clinical picture.

The clinical trajectory of participants involved access to diagnostic and therapeutic intervening resources used by health professionals, such as nursing technicians, nurses, and doctors, of specialized units for the treatment of disease and subsequent obtention to improve and present complications and/or sequelae. To illustrate the integration of the results of the study, Chart 1 is presented, the theoretical framework of summary statements DCS, from the categories of variable of “compulsory notification forms of suspicious of coronavirus disease 2019 - COVID-19 Flu-like Syndromes seized in the first stage (quantitative).”

**Chart 1.** Integration of quantitative and qualitative results. Brazil, 2020-2021

Variables of form mandatory reporting	Central Idea	Discourse of the Collective Subject (Patient experience)
Sociodemographic characteristics	From territorial description to socioeconomic and labor conditions that relate to contagion	[...] *I live in the town, but I need to work in another town. This made me more exposed to the virus. I think I got infected at work or on public transport. Due to the need to keep working to provide for my family, I had no choices. At work, colleagues didn’t always wear a mask and the service didn’t care much about sanitary measures.* (DCS of men who have had COVID-19).
Symptomatology	From the perception of discrete clinical manifestations to the symptomatologic intensification	*[...] I started to feel headaches that became stronger than usual. Symptoms started while I was working. I thought it was something normal, but as the days went by, it got worse. I spent an average of seven days with these pains and then I started to feel pain in my stomach. Then came the tiredness, which increased, and then came the flu-like symptoms such as a runny nose, sore throat, cough, and headaches, which lasted for approximately three days. Then I started to feel chills, fever, body aches, tiredness, lack of energy, fatigue, lack of smell and taste and shortness of breath. I had difficulty sleeping because of the change in breathing and the appearance of nausea.* (DCS of men who have had COVID-19).
Comorbidities	From the conception of good health condition to the appearance of comorbidities	[...] *I had good health; I had no disease. I didn’t lose a night’s sleep, either drink or smoke exaggeratedly. The only change I had was in blood pressure, which I believe is due to my diet.* (DCS of men who have had COVID-19).
Notification	From compulsory notification of the disease to case monitoring	[...] *I suspected Covid-19 and sought the health service. Upon arriving at the health unit, I was referred for an evaluation with a nurse who informed me that she would notify the case. She took me a lot of information about my health status, about the symptoms, when they started, who had contact with me in the past few days and then referred me to undergo the tests. In addition, the nurse told me that I would be monitored by the health team, and in fact this happened, because while I was sick, I received constant calls from the epidemiological surveillance to monitor my situation and to find out if I was complying with the isolation and the care recommended by the health professionals.* (DCS of men who have had COVID-19).
Testing	From initial clinical evaluation to testing for detection of SARS-CoV-2	[...] *I was evaluated by a nursing technician, then by a nurse, and then I took the exam at the COVID-19 care center. Before performing the exam, the nursing technician confirmed some personal information such as my full name, explained to me how the rapid test and the RT-PCR exam in the nasal cavity would be, identified the test with my name and soon after started the procedure, all complying with sanitary requirements.* (DCS of men who have had COVID-19).
Case confirmation	From the receipt of the positive result for COVID-19 to the search for the reasons for the contagion	[...] *I needed to go to the health service to perform the coronavirus detection test and the result was positive. It was a great tension and anguish to have to wait for the result and be stuck inside home [...] I believe I got infected at work because my colleagues didn’t wear masks and I started to relax with the measures of prevention.* (DCS of men who have had COVID-19).
Case evolution	From therapeutic interventions offered by professional teams to self-care measures	[...] *after being evaluated by the nurse, I waited to be evaluated by a doctor, who prescribed me tests and medication and provided me with some information on how I should act in face of COVID-19. During this period, I completed the quarantine and remained at home and taking care so that my family would not be infected. We isolated ourselves. We separated personal belongings such as cutlery and towels and start taking care of domestic hygiene. After using the medicines and following the recommendations for other cares, I improved over the days, and on the date determined by the health service I returned to the nurse and doctor to be reassessed and released to return to normal activities and the abandonment of quarantine. I also tried to eat better, drink plenty of water, teas to improve immunity, sleep well, rest, have faith and positive thinking so as not to bring down my psychological condition.* (DCS of men who have had COVID-19).

Source: Prepared by the authors. Research data.

## DISCUSSION

The analysis of clinical and epidemiological characteristics is essential for the planning and execution of strategic and programmatic actions to promote men’s health and prevent diseases and conditions, especially in critical and complex contexts, such as pandemics. Although the clinical findings have indicated satisfactory clinical outcomes - reduced mortality and high cure rate, assuming mild forms of the disease in the investigated period, the data indicate a growing number of investigated cases. This represents the Brazilian and international scenario and indicates that men are more vulnerable to infection and suffering from COVID-19 and are worthy of attention from health professionals and managers.

Understanding the clinical and epidemiological characteristics of the male public in research on COVID-19 contributes to the early identification and overview of the reasons that indicate why men are getting sick. The discursive findings reported by men strengthen the understanding of health and disease, the experiences of the patient, the clinical pathways, the therapeutic itineraries, barriers and critical routes adopted by men, and weaknesses in healthcare in the pandemic context, which makes it an effective integration methodology to be employed in the production of evidence for the planning and management of healthcare.

Unlike the findings in our study, the clinical profile of the male population with COVID-19 has indicated a high number of complications resulting from impairment by severe forms of the disease, greater need and length of hospital stay, development of SARS, and death.^17^ ^-^ ^18^ It is necessary to point out that the profile of men surveyed involved the residents of a small town located in the semi-arid Bahia, a region with significant rural extension, which can indicate the presence of protective factors, such as healthy lifestyle habits, reduction of urban exposure, work, and other stressors, as well as the presence of satisfactory health behavior and perception of health.

 In our study, young adult men and those with self-reported mixed race/color were the most affected by COVID-19. In the investigated period, vaccination had not yet started in the city, which implies greater exposure of the target population, mainly because in Brazil, the vaccination schedule for COVID-19 includes the young adult population at non-priority levels. Regarding this difference in the clinical-epidemiological profile, epidemiological bulletins in Brazil pointed out in 2020 the impairment of older men, especially in cases of SARS.^17^

However, a focused study had already shown a growing number of severe infections in younger men with an average age of 40 years^19^ in countries like India.^20^ From 2021, the clinical profile in Brazil began to show a growing number of young men affected by COVID-19 and more severe forms of the disease, especially with the arrival of new variants into the country.^21^

 The work environment and occupational relationship of men who were diagnosed with COVID-19 showed a significant relationship with infection. According to the qualitative findings, this may indicate weaknesses in the adoption of individual and collective protection measures, the low quality of personal protective equipment in work environments, and vulnerabilities in access to work, such as in the use of public transport, in addition to failure of institutional protocols for disease prevention and control, as indicated in the literature on the subject.^22^

Regarding compulsory notification of the disease, the full completion of notification forms is extremely useful in the assessment of groups at higher risk, which could be prioritized in actions to combat and control the epidemic in states and municipalities. Such professional action in health is legally required throughout the Brazilian territory and, through the Epidemiological Surveillance Service, allows the monitoring and follow-up of cases. In addition, it presents a panoramic status of active, suspected, under treatment, cured, and death profiles.

Many small municipalities have greater difficulty carrying out epidemiological surveillance actions because of structural limitations and the small number of professionals involved in the health surveillance system. This can compromise the quality of notifications or lead to underreporting of cases, especially in high-demand situations such as the COVID-19 pandemic.^23^

These potential failures in notification systems may occur at the local or national level, and directly impact the conduct of combating actions and delay the collection of knowledge of epidemics. Whether failures occur as a result of the government, or the management and operation of occupational health teams, they can result in episodes of severe disease proliferation, such as what was seen with the COVID-19 pandemic. ^24^ The integration of quantitative and qualitative findings indicated the possibility of weaknesses in the response time between the day of symptom onset and mandatory notification, which may contribute to a greater spread of the virus.

The exponential increase in new registrations observed corroborates the high number of new clinical cases, giving COVID-19 the status of high transmissibility, even if the estimates of the basic reproductive number reported in the literature vary widely.^24^ Thus, the sustained advance of COVID-19 cases worldwide has been accompanied by the formulation of plans for rapid responses to the spread of the disease, conducted in large part by professional nurses, who develop actions of primary care, epidemiological surveillance in health, and assistance to hospitals, among others.^25^

The direction of COVID-19 prevention and epidemiological control strategies among the male population must consider the sociodemographic profile, such as race/color, social class, and occupation, and clinical profile related to signs and symptoms, complaints, complications and infection pattern.^26^ It is necessary to direct more attention specifically to the adult male population, considering that this population is economically active and widely distributed, and, consequently, is more exposed to SARS-CoV-2. Furthermore, according to Brazilian literature, this population has adhered less to preventive health care^27^ ^,^ ^28^. Even out of the context of high risk for viral transmission, male population was more prevalent in the findings identified in this study.

The increase in the number of home deaths, especially among the elderly, as cited in this study, requires attention from health care managers. It reinforces the importance of ambulatory control of chronic diseases and the need to clarify the safety measures to be adopted by the population regarding the clinical complications unrelated to COVID-19 during the pandemic.

The use of combat strategies focused on health promotion, health education, and communication can be decisive factors in preventing and controlling the spread of SARS-CoV-2 among the male population, considering the pattern of behaviors of greater exposure to the risk of infection, for various reasons. These include construction of masculinity, kind of occupational activity, largest social life in the public environment, incorporated concepts of health and health care, beyond personal beliefs about the health and disease process, social and economic vulnerabilities, level of literacy and health literacy, and difficulties in accessing health services.^26^ ^,^ ^27^ Such actions also need to ensure awareness of the male population regarding sanitary measures and adherence to daily health care.

Government efforts must be directed towards ensuring the reach of COVID-19 epidemic control, with focal investments that reach men more precisely. For this, actions with an impact on health in the workplace are necessary, massive testing drives in places with larger male populations, expansion of opening hours of health services, and investment in social communication, with a gender focus.

Finally, with satisfactory effects of the use of these joint efforts, it is possible to reduce the economic impact on the national health system, ensuring greater sustainability of the system before a crisis context. In addition, this will guarantee increased survival and a reduction in male mortality by COVID-19 and secondary complications to the disease, reducing the burden of services and health professionals and will in turn ensure an early and safe return of non-essential daily activities and promotion of social welfare, with positive effects influencing the culture of health care.

As a limitation of the study, some of the cases had incomplete information documented in the medical records, and the clinical documentation of the patients was not homogeneous. However, this is a common limitation in analytical studies, considering that data generation is clinically oriented and not systematically oriented.

## CONCLUSION

The clinical and epidemiological characteristics of men in the investigation of suspected and confirmed cases of COVID-19 were designed for adult men aged 25 to 29 years, mixed race/color, and who did not work in health care. Compulsory notifications were carried out in the municipality of their residence, with an increase in the period from April to August 2020, representing a peak in notified cases. Fatality and maintenance of home treatment were higher among elderly men, and a progression to cure predominated among adolescent, young, and adult men.

The findings in the data pointed to the causal relations of the COVID-19 infection, contagion and transmission, secondary complications generated by the disease, and combat strategies aimed at the male population in the municipal health network. The individual understanding of the disease explains the clinical markers of COVID-19 expressed by the self-reported syndromic approach as well as the search for healthcare in the public service, the adoption of measures to prevent and control illness, and adoption of the recommended therapies by health professionals.
